# Interactions between GHRH and GABAARs in the brains of patients with epilepsy and in animal models of epilepsy

**DOI:** 10.1038/s41598-017-18416-5

**Published:** 2017-12-22

**Authors:** Shirong Tang, Zhong Luo, Xiaowei Qiu, Yanke Zhang, Xi Lu, Hao huang, Zhongxiang Xu, Zucai Xu

**Affiliations:** 1 Department of Neurology, the Affiliated Hospital of Zunyi Medical College, 149 Dalian Road, Zunyi, Guizhou 563003 China; 2 Department of Neurology, the First Affiliated Hospital of Chongqing Medical University, Chongqing Key Laboratory of Neurology, 1 Youyi Road, Chongqing, 400016 China

## Abstract

Growth hormone releasing hormone (GHRH) has recently been shown to increase the level of γ-aminobutyric acid (GABA) and activate GABA receptors (GABARs) in the cerebral cortex. GABA is an inhibitory neurotransmitter that can inhibit seizures. Does GHRH enhance the inhibitory effect of GABA to prevent epilepsy by increasing the GABA level and activating GABARs? In this study, patients with epilepsy and C57/BL6 mice with epilepsy induced by kainic acid (KA) or pentylenetetrazol (PTZ) served as the research subjects. Western blots were used to observe the differences in GHRH expression between the normal group and the epilepsy group, immunofluorescence was performed to explore the localization of GHRH in the brain, and coimmunoprecipitation was used to observe the interaction between GHRH and GABARs. GHRH expression was significantly increased in both patients with temporal lobe epilepsy (TLE) and in two mouse models induced by KA or PTZ compared with that in the normal groups (P < 0.05 or P < 0.01). GHRH was expressed in neurons in both humans and mice. Additionally, GHRH co-localized with presynaptic and postsynaptic sites of inhibitory neurons. Coimmunoprecipitation confirmed that GHRH interacted with GABAAα1 and GABAAβ2 + 3. GHRH may play an important role in inhibiting seizures by activating GABAARs.

## Introduction

Epilepsy is a common chronic neurological disease caused by the abnormal discharge of neurons in the brain and is characterized by short, rigid, repeated neurological dysfunction^1–3^. Temporal lobe epilepsy (TLE) is the most common type of adult epilepsy^4–6^. According to statistics, approximately 70 million patients are currently diagnosed with epilepsy worldwide^7^. Epilepsy greatly increases the health burdens of patients, their families and society and has gained increasing attention from the public. The 68th World Health Assembly, which was held on May 26, 2015, unanimously approved the resolution “Global Burden of Epilepsy and the Need for Coordinated Action at the Country Level to Address its Health, Social and Public Knowledge Implications”. This resolution urged member states to implement coordinated action against epilepsy and its consequences^8^.

Growth hormone releasing hormone (GHRH) is a peptide hormone synthesized by the hypothalamic arcuate neurons that is released into the pituitary portal system; it promotes the release of anterior pituitary growth hormone^9,10^. GHRH has recently been shown to play important roles in promoting the growth and development of the central nervous system (CNS), consolidating memory function, and preventing depression via action of its downstream factors, such as growth hormone (GH)^11,12^. In addition, GHRH increases γ-aminobutyric acid (GABA) levels and activates GABA receptors (GABARs) in the median preoptic nucleus (MnPN) and the ventrolateral preoptic nucleus (VLPO) of the hypothalamus, promoting non-rapid eye movement (NREM) sleep^13^ and increasing insulin-like growth factor 1 (IGF-1) levels to improve cognitive function^14^.

Epilepsy was previously shown to mainly cause neuronal hyperexcitability in the brain, which results from the imbalance between excitation and inhibition mediated by glutamate and GABA, respectively^15,16^. A reduction in GABAergic interneuron activity weakens its suppressive function, thereby increasing the occurrence of spontaneous seizures and promoting the progression of TLE^17^. Transplantation of GABAergic stem cells into the hippocampi of epileptic rats has been shown to reduce the number and intensity of spontaneous seizures caused by chronic epilepsy^18–20^. Does GHRH increase GABA levels and activate GABARs to protect against epilepsy? We conducted the current study to explore this issue.

## Materials and Methods

### Ethics statement

The study was approved by the Ethics Committee of Zunyi Medical College and Chongqing Medical University according to the Declaration of Helsinki. The methods were complied with the ethical guidelines for the medical and health research involving human subjects as established by the National Institutes of Health and the Committee on Human Research at Zunyi Medical College and Chongqing Medical University. Written informed consent was obtained from all patients or their lineal relatives for the use of tissue in research before surgery. All animal experimental procedures were reviewed and approved by the Ethics Committee of Zunyi Medical College and Chongqing Medical University (Approval no. 0002648). All animal experiments were performed according to the principles outlined in the National Institutes of Health guide (NIH Publications No. 8023, revised 1978). Furthermore, all efforts were made to minimize the number of animals used in the experiment and their suffering.

### Human samples

All clinical samples were randomly selected from our brain tissue bank, which includes 340 tissues from patients with epilepsy and 120 control tissues. In our experiment, samples were collected from 22 patients [13 males and 9 females; mean age 35.23 ± 8.48 years (20–52 years); mean disease course 11.95 ± 6.01 years (5–26 years)] who had undergone resection surgery for intractable epilepsy. All presurgical assessments comprised a detailed history and neurological examination to ensure that these patients were suitable candidates for surgery, as previously described [3]. All patients had taken three or more antiepileptic drugs (AEDs). Twelve histologically normal samples were randomly selected for use as a control group [7 males and 5 females; mean age 32.17 ± 8.31 years (20–52 years)]. The control samples were obtained from patients who had undergone cranial surgery due to increased intracranial pressure caused by head trauma and who had no history of epilepsy, had not been exposed to AEDs and had no apparent signs of CNS disease. Additionally, no significant differences in gender or age were observed between the patients and the control group (P > 0.05). The clinical features of the patients with TLE and the controls are summarized in Tables 1 and 2, respectively.

**Table 1:** Clinical characteristics of TLE patients

Patients	Age (y)	Sex (M/F)	Course (y)	AEDs before surgery	Pathology	Resection tissue
1	34	M	8	CBZ, VPA, PHT, TPM	NL	TNl
2	46	M	15	LTG, CBZ, VPA, PHT	NL	TNl
3	38	F	11	OXC, PB, TPM, PHT	NL	TNr
4	32	M	6	CBZ, TPM, PHT, VPA	NL	TNr
5	27	F	13	LTG, CBZ, PB, PHT	Gliosis	TNr
6	40	F	8	CBZ, VPA, TPM	Gliosis	TNl
7	25	F	5	PB, CBZ, VPA, PHT	NL	TNr
8	33	M	12	LTG, CBZ, PHT, VPA	NL	TNr
9	29	M	10	CBZ, VPA, PB, PHT	Gliosis	TNl
10	35	F	14	CBZ, VPA, TPM, LEV	NL	TNl
11	43	M	20	OXC, VPA, PHT, LTG, PB	Gliosis	TNl
12	22	M	6	VPA, CBZ, LTG, PHT	NL	TNr
13	20	M	5	OXC, VPA, CBZ, PHT	Gliosis	TNl
14	49	F	25	LTG, TPM, VPA, LEV	NL,Gliosis	TNl
15	32	M	7	VPA, CBZ, PB, LTG	NL	TNl
16	41	M	13	LTG, PB, LTG, OXC	Gliosis	TNr
17	52	M	26	LEV, CBZ, VPA, PHT, TPM	Gliosis	TNr
18	37	F	9	LTG, VPA, TPM, PHT	NL	TNl
19	26	M	7	OXC, VPA, CBZ, PHT	Gliosis	TNl
20	36	F	15	PB, VPA, TPM, LEV	NL	TNl
21	43	F	18	VPA, PB, TPM, LTG	Nl	TNr
22	33	M	10	VPA, PB, TPM, LEV	Gliosis	TNr

M, male; F female; y, year; AEDs, antiepileptic drugs; CBZ, carbamazepine; GBP, gabapentin; LEV, levetiracetam; LTG, lamotrigine; OXC, oxcarbazepine; PB, phenobarbital; PHT, phenytoin; TPM, topamax; VPA, valproic acid; TN, temporal neocortex; l, left; r, right; NL, neuron loss.

**Table 2:** Clinical characteristics of the control patients

Patients	Age (y)	Sex (M/F)	Resection tissue	Pathology
1	30	M	TNr	N
2	38	M	TNl	N
3	26	M	TNl	N
4	29	F	TNl	N
5	37	M	TNr	N
6	42	F	TNr	N
7	22	F	TNl	N
8	35	F	TNr	N
9	48	M	TNr	N
10	32	M	TNr	N
11	19	F	TNl	N
12	28	M	TNr	N

F, female; M, male; y, year; TN, temporal neocortex; l, left; r, right; N, normal.

### Mouse models of epilepsy

All adult male C57/BL6 mice weighing 24–28 g were obtained from the Experimental Animal Center of Chongqing Medical University, China, and were randomly divided into a normal control group and an experimental group. All animals were housed in cages with a 12/12 h light/dark cycle and other standard conditions, including a room temperature of 23 ± 1 °C and access to food and water ad libitum. The pentylenetetrazol (PTZ) kindling model was developed using a previously reported protocol^21^. Mice were intraperitoneally injected with PTZ (35 mg/kg, Sigma-Aldrich, St. Louis, MO, USA) once every other day for a total of 15 injections (from day 1 to day 30). After each injection, all animals were immediately observed for 30 min. The evoked seizures were scored using the criteria described by Racine (1972). Mice with at least three consecutive seizures with a score 4 or 5 were considered fully kindled. The kainic acid (KA)-induced chronic epilepsy model was generated as previously described^22,23^. Briefly, stereotaxic injections into the dorsal blade of the CA1 area were made at the following coordinates with respect to the bregma: anteroposterior-1.8 mm, mediolateral-1.5 mm, and 1.5 mm below the dura. Mice were deeply anesthetized and unilaterally injected with 1.0 nmol of KA (Sigma-Aldrich Co., St. Louis, MO, USA), and 50 nl saline were injected into the hippocampus in the contralateral hemisphere. We injected KA for 1 min with a 0.5 μl microsyringe (Hamilton, Reno, NV)^17^. In addition, the microsyringe was left *in situ* for 5 min after the injection and was then withdrawn slowly to reduce backflow along the injection trace.

### *In vivo* multichannel electroencephalogram (EEG) recordings

EEG recordings were captured from the different groups (control group and epilepsy group) in the chronic phase of the KA-induced epilepsy model. We implanted a microwire array into the hippocampus of each mouse and performed multichannel EEG recordings *in vivo*. We used an OmniPlex® Dneural Data Acquisition System (Plexon, Dallas, TX, USA) to record EEGs, and defined an electrophysiological seizure as a seizure with a high frequency (>5 Hz) and high amplitude (>2 times the baseline) that lasted for more than 5 s.

### Tissue preparation

#### Humans

All human samples were collected from patients in the operating room, immediately frozen in liquid nitrogen and stored at −80 °C until they were used for Western blotting. The remaining samples were sectioned at a 10 μm thickness and stored at −20 °C for immunofluorescence analysis.

#### Mice

For the immunofluorescence analysis, the brain tissues were fixed with 4% paraformaldehyde and then successively incubated in 20% and 30% sucrose in PBS for 24 h. The tissues were then sectioned at 10 µm at −20 °C for the laser confocal microscopy analysis. For Western blotting, the neocortex and hippocampus were flash-frozen in liquid nitrogen and then stored at −80 °C until further use.

### Immunofluorescence staining

Immunofluorescence staining was performed using the method described in our previous publication^4,24^. The primary antibodies were: rabbit anti-GHRH (1:100, catalog No: ab187512, Abcam, Cambridge, MA, USA), mouse anti-glial fibrillary acidic protein (GFAP) (1:50, catalog number BM0055; Boster Bioengineering, Wuhan, China), mouse anti-Gephyrin (1:50, Abcam, Cambridge, MA, USA), guinea pig anti-microtubule-associated protein 2 (MAP2) (1:200, Sysy, Goettingen, Germany), mouse anti-glutamate decarboxylase 67 (GAD67) (1:100, Abcam, Cambridge, MA, USA), and guinea pig anti-vesicular GABA transporter (VGAT) (1:200, Sysy, Goettingen, Germany). The secondary antibodies were: an Alexa Fluor 488-conjugated goat anti-rabbit IgG antibody (1:50, Zhongshan Golden Bridge, Inc., Beijing, China), Alexa Fluor 594-conjugated goat anti-mouse IgG antibody (1:200, Zhongshan Golden BridgeInc., Beijing, China), and Alexa Fluor 633-conjugated goat anti-guinea pig IgG antibody (1:50, Abcam, Cambridge, MA, USA). Finally, the samples were treated with 4′,6-diamidino-2-phenylindole dihydrochloride (Sigma, St. Louis, MO, USA) for 5 min to identify the nuclei. Immunofluorescently labeled sections were examined with a laser scanning confocal microscope (Leica Microsystems, Wetzlar, Germany) and an Olympus IX 70 inverted microscope (Olympus America, Melville, NY, USA).

### Western blot and coimmunoprecipitation

#### Western blot

The tissue samples were collected from humans and mice for Western blot analysis using a previously described protocol^4,25^. Briefly, total proteins were extracted according to the manufacturer’s protocol (Keygen Biotech, Nanjing, China). SDS-PAGE gels (5% stacking gel; 10% separating gel) were used to separate total protein lysates (40 μg per lane) and electrophoretically transferred to polyvinylidene fluoride membranes (PVDF) (Millipore Corporation, USA). PVDF membranes were incubated with 5% nonfat milk for 1 h at room temperature (RT) to prevent non-specific binding. Later, the membranes were incubated with rabbit anti-GHRH (1:500, catalog No: ab187512, Abcam, Cambridge, MA, USA) and anti-GAPDH (1:3,000, Proteintech, Wuhan, China) antibodies overnight at 4 °C. The secondary antibody (peroxidase-conjugated goat anti-rabbit IgG (1:3,000, Proteintech, Wuhan, China) was incubated with the PVDF membranes for 1 h at RT on the next day. Enhanced chemiluminescence (ECL) reagent (Thermo, Marina, CA, USA) and a Fusion FX5 image analysis system (Vilber Lourmat, Marne-la-Vallée, France) were used to visualize the bands. Finally, Quantity One software (Bio-Rad, CA, USA) was used to measure the resulting optical density (OD) values, which were normalized to GAPDH expression.

#### Coimmunoprecipitation

Coimmunoprecipitation was performed according to the method used in our previous publication^4^. Protein extracts from mouse hippocampal tissues were homogenized and then mixed with immunoprecipitation (IP) lysis buffer. Equal amounts of proteins were incubated with 2 μl of rabbit IgG (Abcam, Cambridge, MA, USA) as a polyclonal isotype control, 2 μl of GHRH (Abcam, Cambridge, MA, USA), 4 μl of GABAAα1, or 4 μl of GABAAβ2 + 3 antibodies (Proteintech, Wuhan, China) overnight at 4 °C; then, Protein A/G agarose beads (20 μl; Santa Cruz Biotechnology, Dallas, TX, USA) were incubated with the samples for 2 h at 4 °C. The protein-bead complexes were washed five times and pelleted by centrifugation. Next, the samples were mixed with 1× loading buffer and heated for 5 min at 95 °C. Finally, the samples were used for Western blots with the same series of antibodies described above.

### Statistical analysis

We analyzed the data using Student’s t-test to evaluate the differences between two groups (two-independent samples, normal distribution and equal variances) with SPSS18.0 software (SPSS, Inc., Chicago, IL, USA). Each experiment was repeated three times. The χ^2^ test was used to compare the gender-specific differences between patients with TLE and controls. P < 0.05 or P < 0.01 was considered a statistically significant difference. The data are presented as the Mean ± SEM. Data were also analyzed using GraphPad Prism software (GraphPad Prism Software, San Diego, CA, USA).

### Data availability

The datasets generated during and/or analysed during the current study are available from the corresponding author on reasonable request.

## Results

### Clinical characteristics

In the study, all patients in the TLE group had a seizure recurrence history of at least 5 years and had taken three or more antiepileptic drugs (AEDs). In addition, the TLE group had a mean age of 35.23 ± 8.48 years (20–52 years) and consisted of 22 patients (13 males and 9 females); the average duration of epilepsy was 11.95 ± 6.01 years (5–26 years) (Table 1). The control group had a mean age of 32.1 7 ± 8.31 years (20–52 years) with 7 men and 5 women (Table 2). No significant differences in age and gender were observed between the TLE and control groups (P > 0.05).

### Increased GHRH expression in patients with TLE

**Figure 1:**
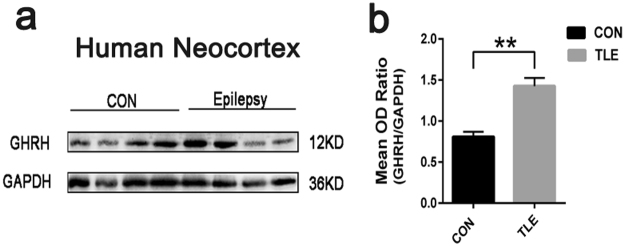
Increased GHRH expression in patients with TLE. (**a)** Representative Western blots showing GHRH expression in patients with TLE and controls. (**b)** The mean OD ratio for GHRH was significantly increased in the TLE group (n = 22) compared to that in the control group(n = 12) (1.43 ± 0.10 vs. 0.81 ± 0.06 **P < 0.01). The bars indicated the mean ± SEM.

First, we performed Western blotting to investigate GHRH expression levels in the cortices of patients with epilepsy (n = 22) and controls (n = 12); GAPDH expression was assessed as an internal loading control and was present as a 36 kDa band (Fig. 1a). In our experiment, a 12 kDa GHRH band was detected (Fig. 1a), and the mean OD ratio for GHRH was significantly increased in the TLE group (1.43 ± 0.10) compared to that in the control group (0.81 ± 0.06) (Fig. 1a). GHRH expression levels were normalized by calculating the OD ratio of GHRH to GAPDH (GHRH/GAPDH). These values were significantly different (P < 0.01) (Fig. 1b). All data are presented as the Mean ± SEM.

### Increased GHRH expression in mouse epilepsy models

**Figure 2:**
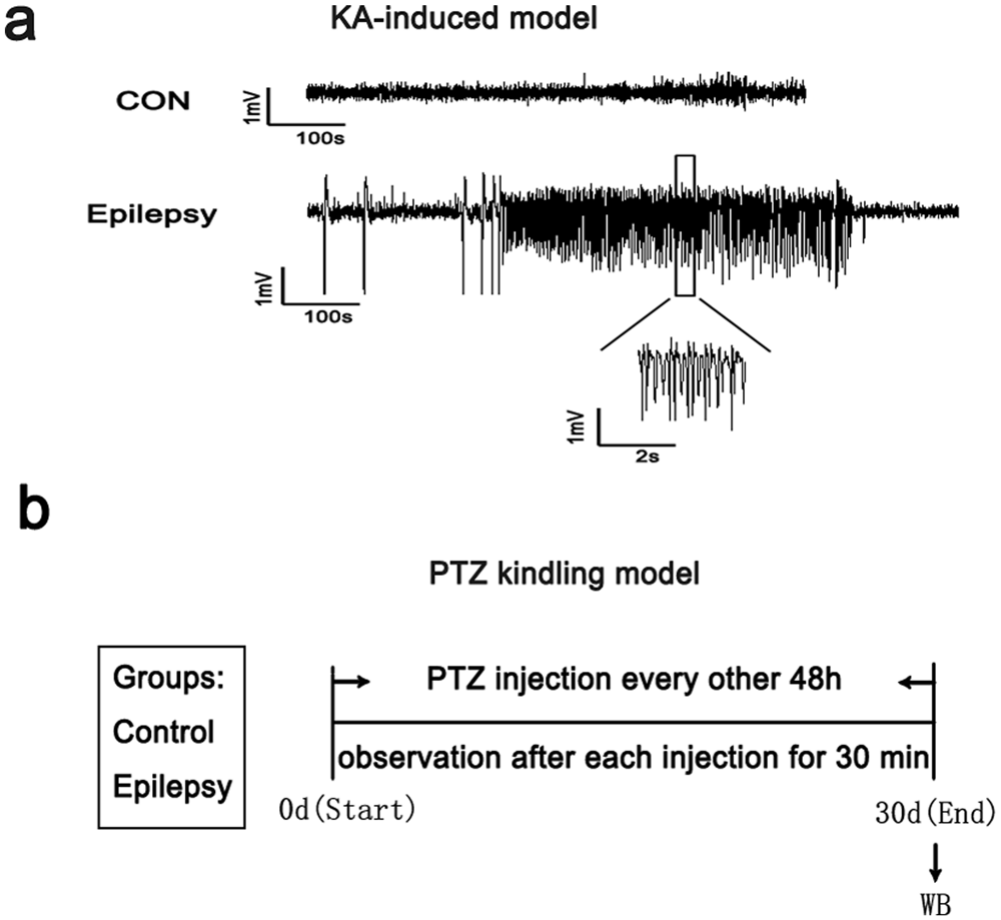
Classification of the animal groups in the two mouse models (n = 5 per group). **(a)** Representative EEG recordings from epileptic and control mice in the KA-induced model. The EEG of the epilepsy group exhibited more frequent bursts of spikes and sharp waves than were observed for the control group. (**b)** Classification of the animal groups and the process used to initiate the PTZ kindling model.

**Figure 3:**
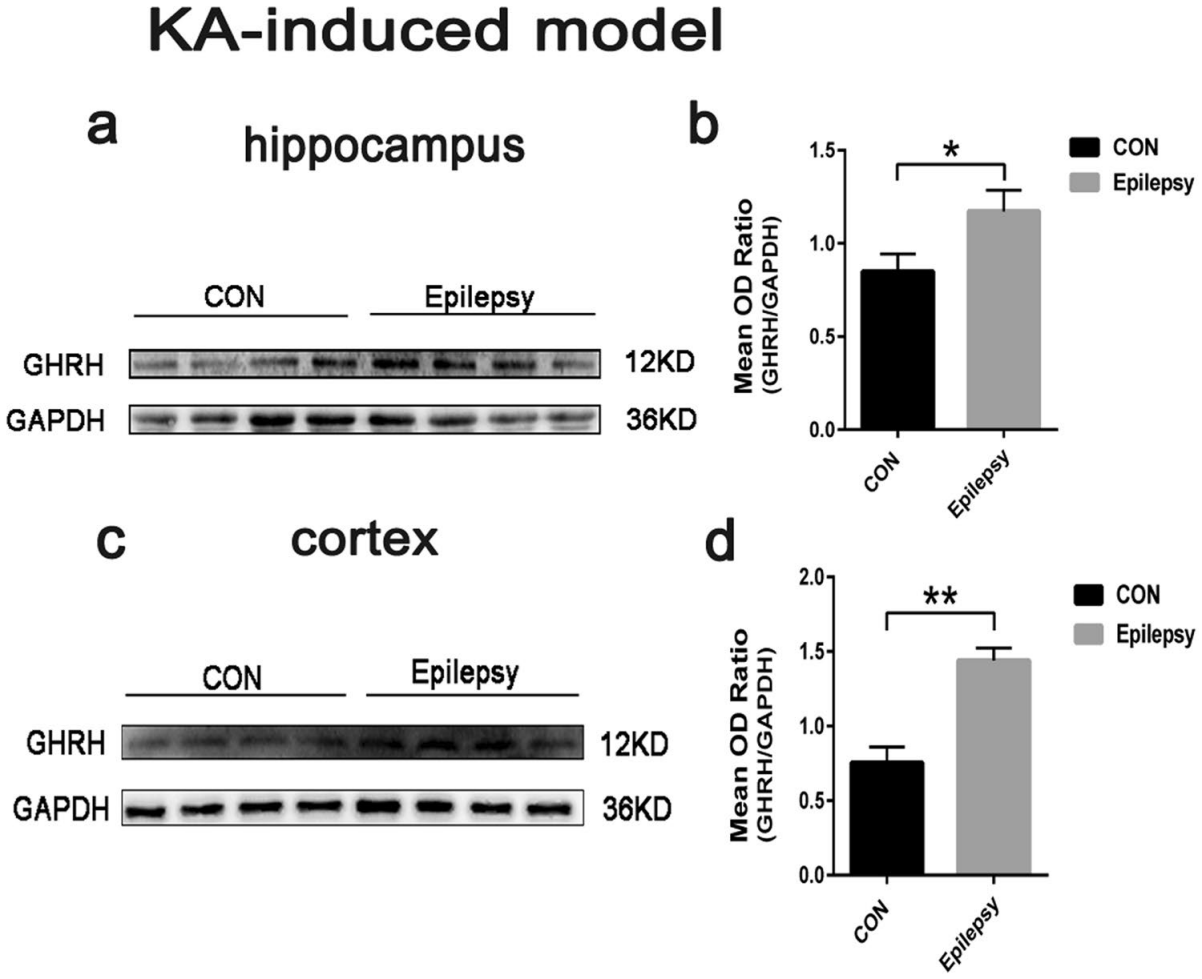
Increased GHRH expression in the KA-induced model. (**a**,**c)** Representative Western blots showing the expression of GHRH in the hippocampus and cortex of the mouse model of KA-induced epilepsy and control mice. (**b**,**d)** Mean OD ratios for GHRH were significantly increased in the hippocampus and cortex of the mouse model of KA-induced epilepsy compared with those in the control group(n = 5 per group). (hippocampus: 1.17 ± 0.11 vs. 0.85 ± 0.09 *P < 0.05; cortex: 1.44 ± 0.08 vs. 0.75 ± 0.11 **P < 0.01). Data indicated the mean ± SEM.

Next, we examined GHRH expression in mouse models of epilepsy induced by KA (Fig. 2a) and PTZ (Fig. 2b) using Western blotting. The evoked seizures were scored in accordance with the criteria reported by Racine (1972). Mice with at least three consecutive seizures with scores of 4 or 5 were considered fully kindled. Moreover, EEG recordings were captured from both the control and epilepsy group in the chronic phase of the KA-induced model (Fig. 2a). Similar to the human samples, the GHRH expression levels were significantly increased in the hippocampus and cortex of the epileptic mice compared to the levels in control mice. In the KA-induced epilepsy mouse model, the GHRH expression levels in the hippocampus and cortex of epileptic mice were 1.17 ± 0.11 and 1.44 ± 0.08 compared to 0.85 ± 0.09 and 0.75 ± 0.11 in the control mice, respectively (Fig. 3a,c). All data are shown as the mean ± SEM.

**Figure 4:**
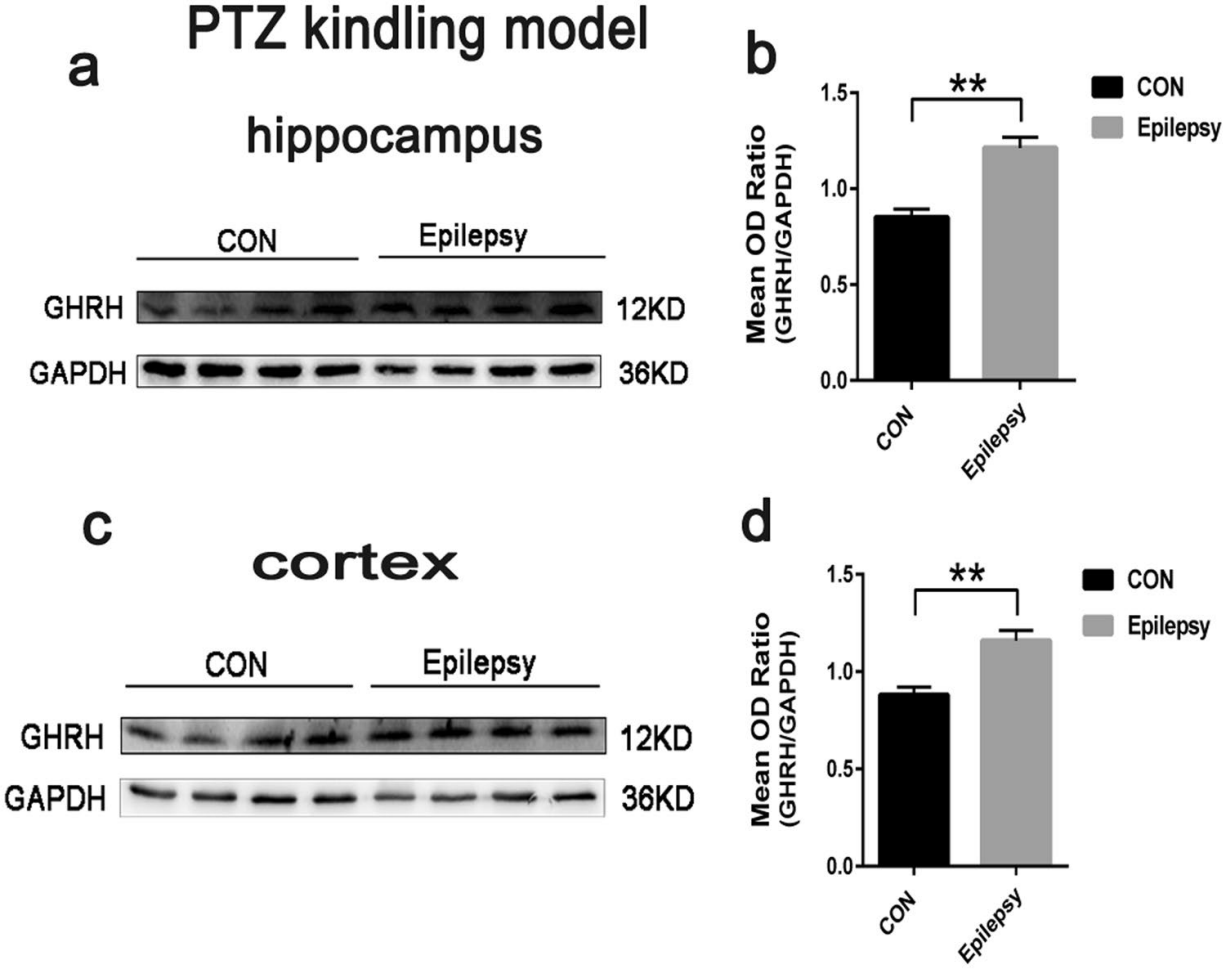
Increased GHRH expression in the PTZ kindling model. (**a**,**c)** Representative Western blots showing GHRH expression in the hippocampus and cortex of the mouse model of PTZ-kindled epilepsy and control mice. (**b**,**d)** Mean OD ratios of GHRH were significantly increased in the hippocampus and cortex of the mouse model of PTZ-kindled epilepsy compared to those in the control group(n = 5 in each group). (hippocampus: 1.21 ± 0.05 vs. 0.85 ± 0.04 **P < 0.01; cortex: 1.16 ± 0.05 vs. 0.88 ± 0.04 **P < 0.01). Data are expressed as the mean ± SEM.

Similarly, in a mouse model of PTZ-kindled epilepsy, the GHRH expression levels in the hippocampus and cortex of epileptic mice were 1.21 ± 0.05 and 1.16 ± 0.05 compared with 0.85 ± 0.04 and 0.88 ± 0.04 in the control mice, respectively (Fig. 4a,c). GAPDH was also used as an internal control, and the mean OD value of GHRH was normalized to GAPDH (GHRH/GAPDH). These values were significantly different (P < 0.05 or P < 0.01) (Figs 3b,d and 4b,d). Data are expressed as the mean ± SEM.

### Localization of GHRH in the human neocortex and in the mouse hippocampus and cortex

**Figure 5:**
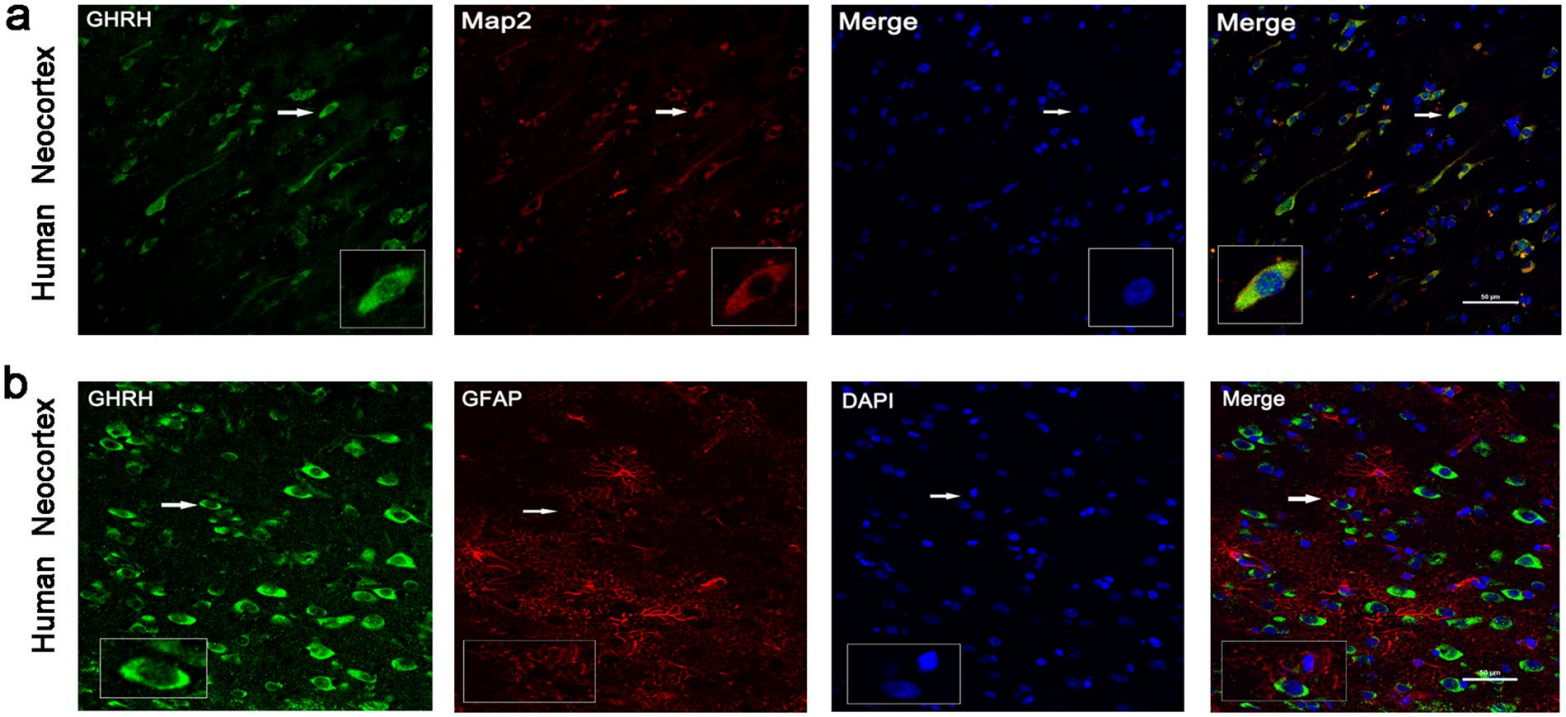
Localization of GHRH in the human neocortex. (**a**) GHRH (green) co-localized with MAP2-positive (red) neurons in the human neocortex. (**b**) GHRH (green) and GFAP (red) were not co-expressed in astrocytes in the human neocortex. DAPI (blue) indicates the cell nuclei. The white squares indicate positive cells, and the scale bar represents 50 μm.

**Figure 6:**
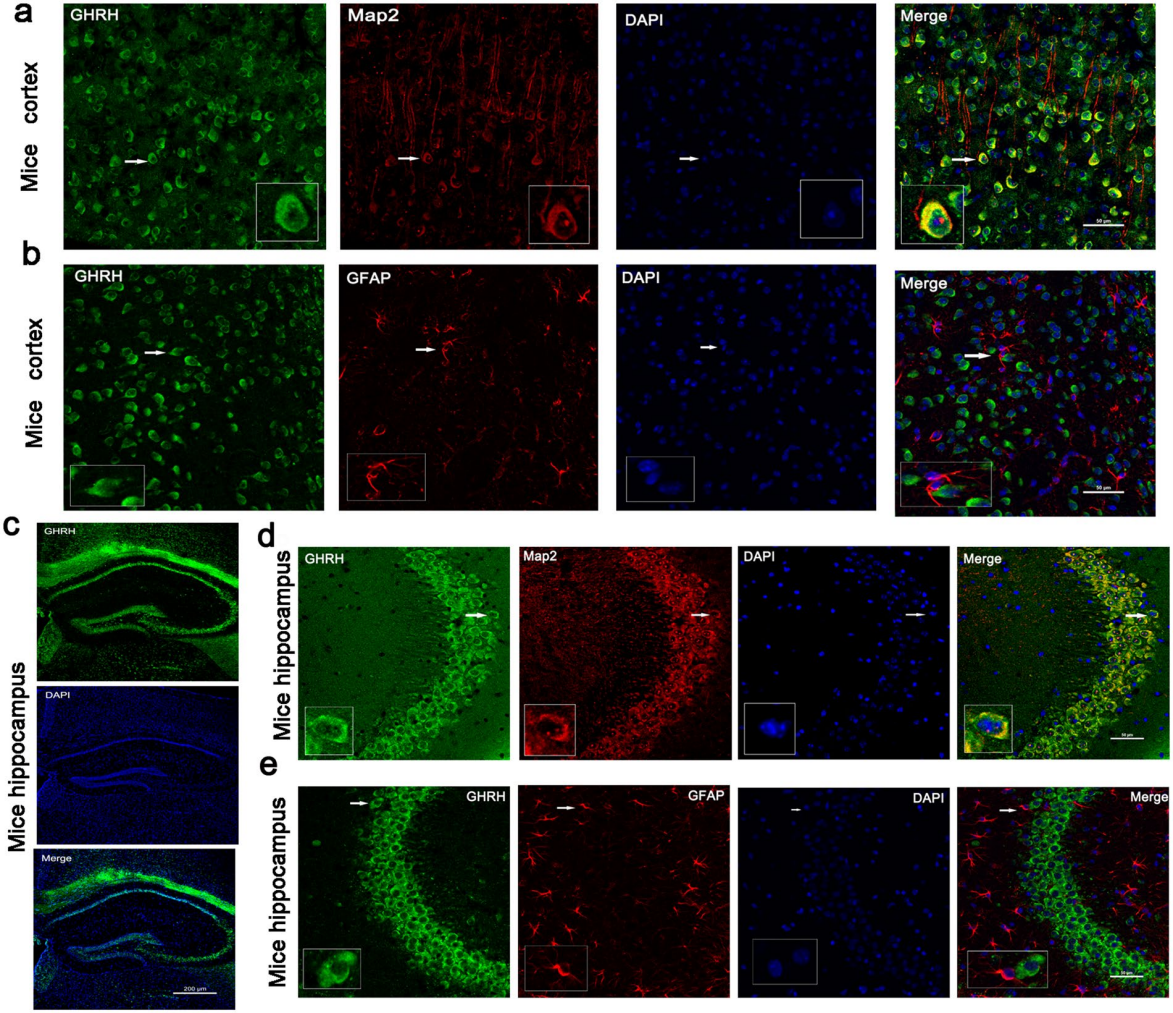
Localization of GHRH in the mouse cortex and hippocampus. (**a**,**d)** GHRH (green) co-localized with MAP2-positive (red) neurons in the mouse cortex and hippocampus. (**b**,**e)** GHRH (green) and GFAP (red) were not co-expressed in astrocytes in the mouse cortex and hippocampus. DAPI (blue) indicates the cell nuclei. The white squares indicate positive cells, and the scale bar represents 50 μm. (**b**) GHRH (green) was localized in both the cortex and the whole hippocampus, and cell nuclei were counterstained with DAPI (blue). Scale bar = 200 μm.

**Figure 7:**
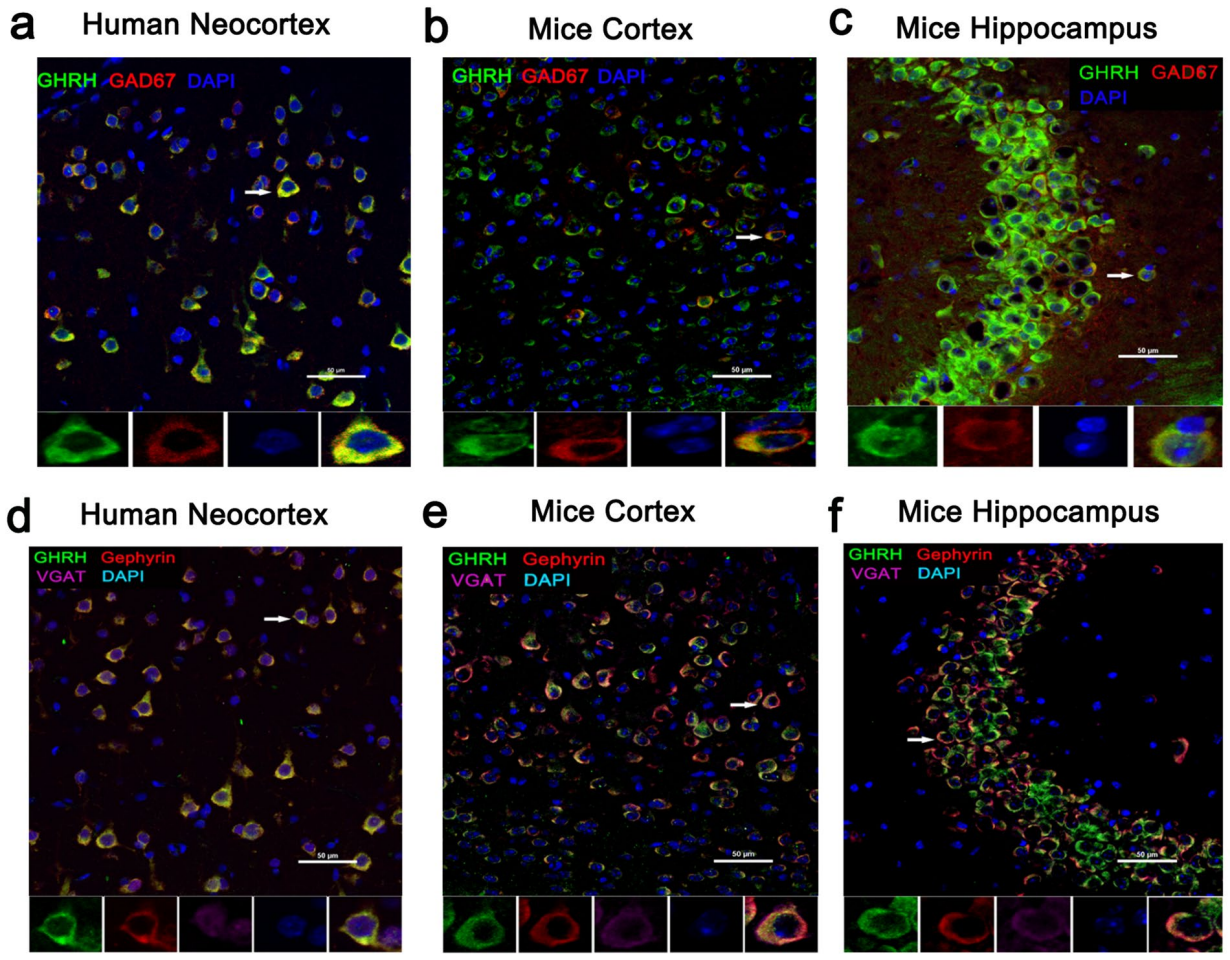
Localization of GHRH in inhibitory neurons and synapses. (**a)** Immunofluorescence staining showing that GHRH (green) co-localized with GAD67-positive (red) inhibitory neurons in the human neocortex; and DAPI (blue) indicates the cell nuclei. (**b**,**c)** Representative immunofluorescence staining showing that GHRH (green) overlapped with GAD67-positive (red) inhibitory neurons and DAPI (blue) in the mouse hippocampus and cortex. (**d)** Immunofluorescence staining showing that GHRH (green) overlapped with VGAT (purple, a marker of Inhibitory presynaptic neurons) and Gephyrin (red, a marker of inhibitory postsynaptic neurons) in the human neocortex; and DAPI (blue) indicates the cell nuclei. (**e**,**f)** Representative immunofluorescence staining showing that GHRH (green) was co-expressed with VGAT (purple) and Gephyrin (red) in the mouse hippocampus and cortex. Cell nuclei were counterstained with DAPI (blue). The arrows and white squares indicate positive cells, and the scale bar represents 50 μm.

Immunofluorescence was performed on cortical tissues obtained from adult human brains to examine the localization of GHRH. GHRH (green) was co-expressed with MAP2 (red, a marker of dendrites) in the cytoplasm of neurons (yellow) (Fig. 5a), but was not co-expressed with GFAP (red, a marker of astrocytes) in the human neocortex (Fig. 5b). Furthermore, we investigated the localization of GHRH in the mouse hippocampus and cortex. GHRH (green) was localized in both the cortex and the whole hippocampus (Fig. 6a,d), and the expression overlapped with MAP2-positive (red) neurons. However, GHRH (green) was not co-localized with GFAP (red) in astrocytes (Fig. 6b,e). In addition, we further studied the localization of GHRH in specific neurons and subcellular structures in the human neocortex. GHRH was co-expressed with GAD67 (red, a marker of inhibitory neurons) (Fig. 7a) and VGAT (purple, a marker of presynaptic specializations from inhibitory neurons) and Gephyrin (red, a marker of inhibitory specializations from postsynaptic neurons) (Fig. 7d). Finally, we obtained consistent results in the mouse hippocampus and cortex. GHRH staining overlapped with GAD67 (red) (Fig. 7b,c), VGAT (purple) and Gephyrin (red) staining (Fig. 7e,f).

### Interaction between GHRH and GABAARs

**Figure 8:**
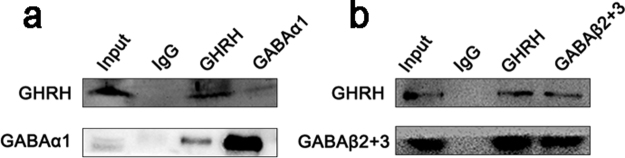
Interaction between GHRH and GABAARs. Coimmunoprecipitation of GABAAα1 **(a)** and GABAAβ2 + 3 **(b)** with anti-GHRH antibodies, which was verified by the reciprocal coimmunoprecipitation of GHRH with anti-GABAAα1 **(a)** and anti-GABAAβ2 + 3 antibodies **(b)**.

Immunoprecipitations were conducted using mouse hippocampal tissues to further examine the interactions in which GHRH participates in inhibitory synapses in the hippocampus. GABAAα1 (Fig. 8a) and GABAAβ2 + 3 (Fig. 8b) were coimmunoprecipitated with anti-GHRH antibodies, indicating that GHRH interacted with GABAARs. This interaction was verified by the reciprocal coimmunoprecipitation of GHRH with anti-GABAAα1 (Fig. 8a) and anti-GABAAβ2 + 3 antibodies (Fig. 8b).

## Discussion

The primary finding of this study is that GHRH expression was significantly increased in the brains of both patients with TLE and in two mouse models of epilepsy induced by either KA or PTZ. Second, most of the GHRH-positive cells co-expressed neuronal markers, but not glial cell markers, in both human and mouse tissues. In addition, GHRH co-localized with presynaptic and postsynaptic inhibitory neurons. Interestingly, we confirmed that GHRH and GABAARs interacted. Therefore, we propose that GHRH may exert an antiepileptic effect.

GHRH has been shown to increase GABA levels and activate GABARs to promote NREM sleep, which contributes to the regulation of sleep by GH. Peterfi Z. *et al*. discovered both NREM sleep amount and double-labeled Fos + glutamic acid decarboxylase (GAD) cell counts in the MnPN and VLPO were significantly increased in rats that received intracerebroventricular injection of GHRH compared with saline-treated rats. While the opposite results were obtained in the octreotide (somatostatin analog OCT) and the high dose of GHRH antagonist-treated groups. All the results identified GABAergic neurons as potential targets of the sleep-regulatory actions of GHRH in the MnPN and VLPO^13^. Furthermore, a study using an amyloid precursor protein (APP) transgenic mouse model showed that mice express GHRH-dependent factors that protect against amyloid β Aβ) deposition. GHRH also increases the level of IGF-1, thereby collectively improving Alzheimer’s disease (AD)-and obstructive sleep apnea (OSA)-related cognitive dysfunction^14,26^. In addition, a clinical research unit at the University of Washington School of Medicine conducted a randomized, double-blind, placebo-controlled study that administered a 1 mg subcutaneous injection of a human GHRH analogue daily. After 20 weeks, GABA levels were increased in the brain (P < 0.04), and IGF-1 levels were positively correlated with GABA levels (r = 0.47; P = 0.001). However, myo-inositol (MI, an AD-related marker) levels decreased (P = 0.002), which may be one of the mechanisms by which GHRH and IGF-1 improve cognitive function during normal aging^11^.

The GABAA receptor (GABAAR) is a part of the ligand-gated ion channel complex that mediates the opening of the chloride channel. GABAARs are the most important part of inhibitory neurotransmission in the brain and are composed of the following subunits: α1–6, β1–3, γ1–3, δ, ε, π, θ, and ρ1–3, among which, the α1–3, β2–3 and γ2 subunit compositions of five dimers play a major role^6,27^. Changes in the expression of these subunits and alterations in the function of the receptor induced by mutation, tumors or hypoxia may interfere with inhibitory transmission by GABAARs, resulting in epilepsy^15,28^. For example, the alpha 1 subunit D219N mutation has been identified in patients with idiopathic generalized epilepsy^29^, and an A322D mutation was detected in patients with juvenile myoclonic epilepsy^30^. Fluoxetine has been shown to antagonize brain injury by increasing GABAAR levels in the dentate gyrus and CA1-CA2 areas of rats with pilocarpine-induced epilepsy^31^. As shown in our previous studies, ubiquitin-like protein 1 (plic-1), which is enriched in inhibitory synapses and is associated with GABAARs and the ubiquitin proteasome system (UPS), activates GABAARs, thereby inhibiting seizures^32^.

In this study, GHRH expression was first assessed by Western blotting and was significantly increased both in patients with TLE and in two mouse models of epilepsy induced by either KA or PTZ. Moreover, immunofluorescence staining revealed the co-localization of most of the GHRH-positive cells with neurons, but not glial cells, in both humans and mice. We incubated sections with antibodies against inhibitory neurons, inhibitory synaptic markers (GAD67, VGAT and Gephyrin) and GHRH and examined their immunofluorescence localization to further determine whether GHRH was related to inhibition. GHRH co-localized with presynaptic and postsynaptic inhibitory neurons. Then, coimmunoprecipitations were performed to examine whether GHRH interacted with GABAR at inhibitory synapses and participated in the inhibitory function. Surprisingly, coimmunoprecipitation experiments confirmed interactions between GHRH and GABAAα1 and GABAAβ2 + 3. Therefore, we propose that GHRH participates in anti-epileptic processes by increasing GABA levels and activating GABAARs. However, the results in this study are descriptive and do not provide evidence showing that GHRH contributes to an antiepileptic effect. Further studies are needed to test whether GHRH directly interacts with GABAARs to further activate them, thereby enhancing the inhibitory effect to prevent epilepsy.

## Electronic supplementary material

Supplementary Information 
