# Supplementary material for: Interactions between GHRH and GABAARs in the brains of patients with epilepsy and in animal models of epilepsy
Source: Sci Rep. 2017 Dec 22;7:18110. doi: 10.1038/s41598-017-18416-5 (PMC5741719; doi:10.1038/s41598-017-18416-5)

Interactions between GHRH and GABAARs in the brains of patients with epilepsy and in animal models of epilepsy

Shirong Tang[1] Zhong Luo[1] Xiaowei Qiu[1] Yanke Zhang[2] Xi Lu[2] Hao huang[1] Zhongxiang Xu[1] Zucai Xu[1]

Human Neocortex

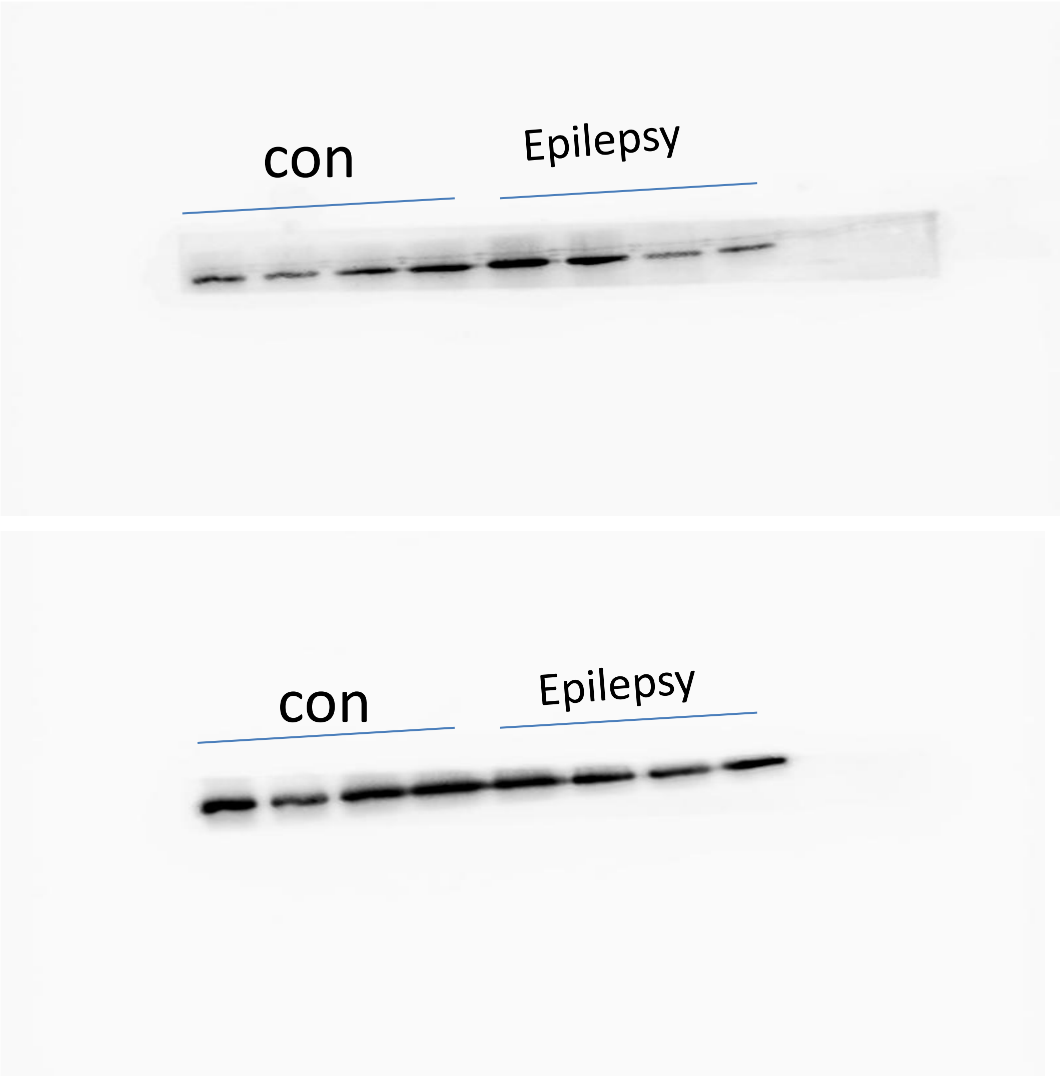

Interactions between GHRH and GABAARs in the brains of patients with epilepsy and in animal models of epilepsy

Shirong Tang[1] Zhong Luo[1] Xiaowei Qiu[1] Yanke Zhang[2] Xi Lu[2] Hao huang[1] Zhongxiang Xu[1] Zucai Xu[1]

KA-induced model

hippocampus

GHRH

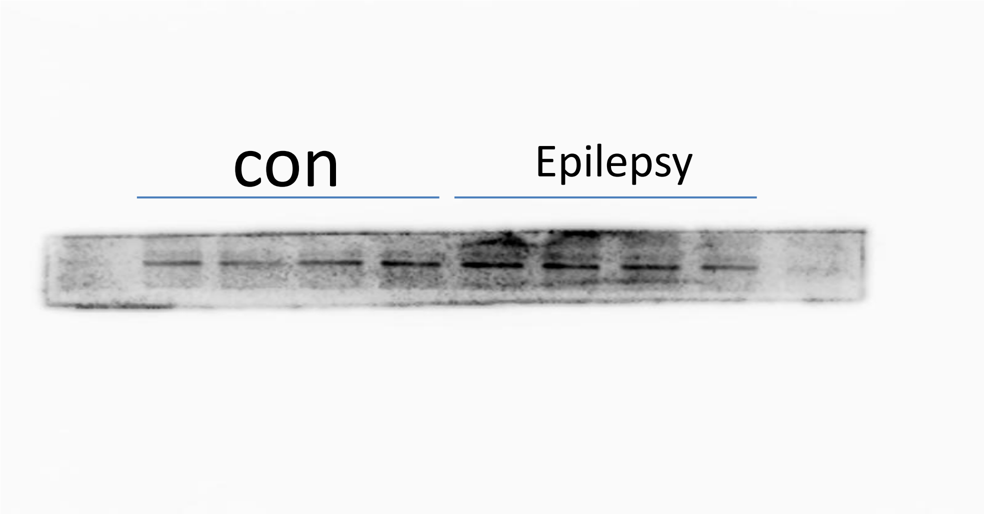

GAPDH

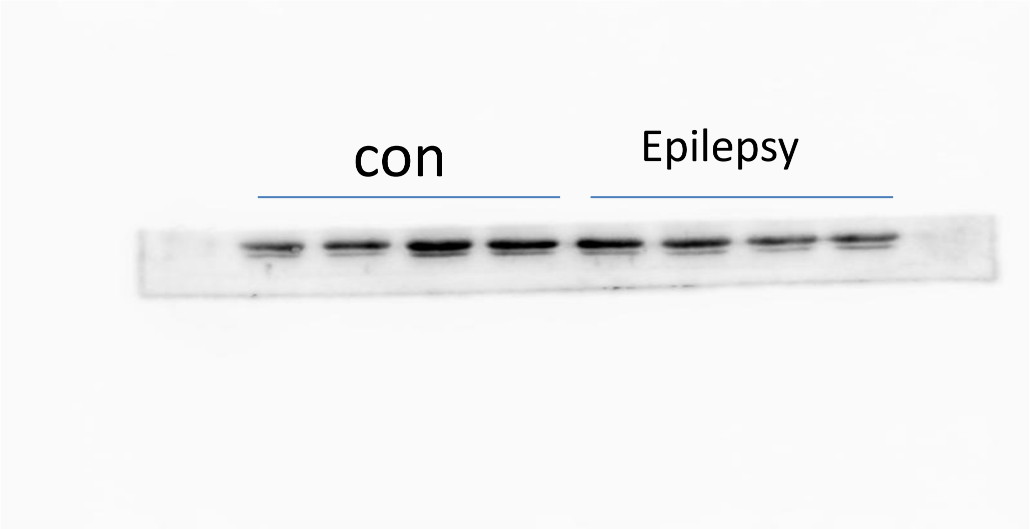

Interactions between GHRH and GABAARs in the brains of patients with epilepsy and in animal models of epilepsy

Shirong Tang[1] Zhong Luo[1] Xiaowei Qiu[1] Yanke Zhang[2] Xi Lu[2] Hao huang[1] Zhongxiang Xu[1] Zucai Xu[1]

KA-induced model

cortex

GHRH

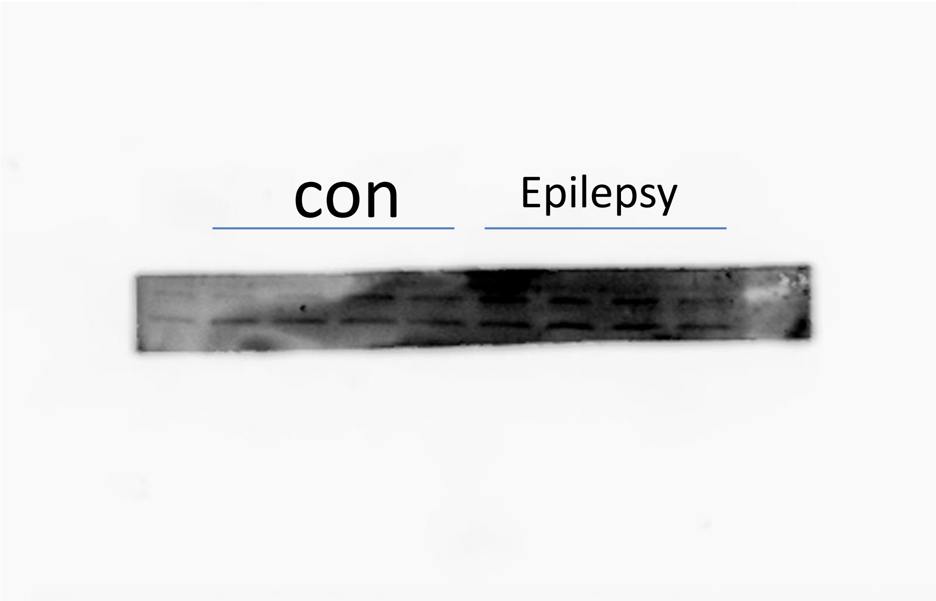

GAPDH

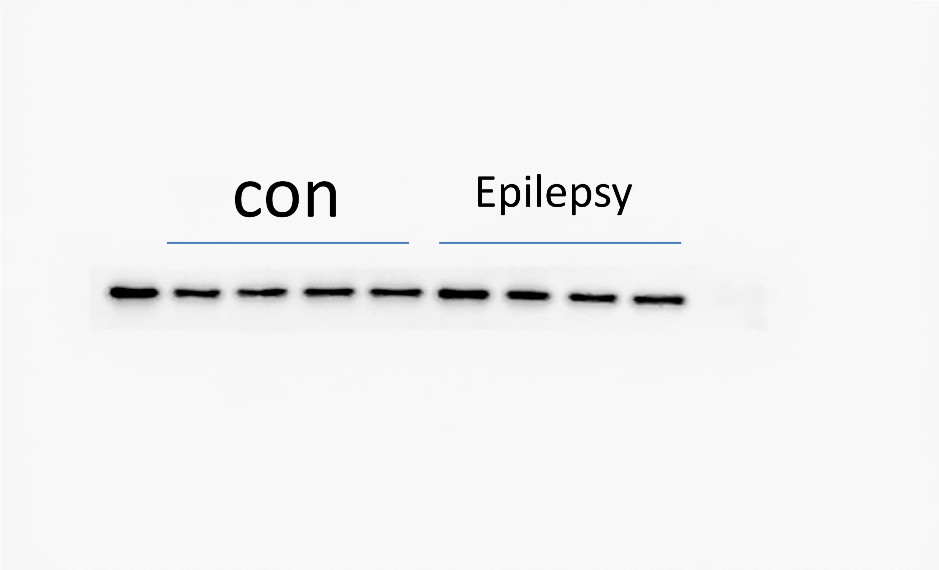

Shirong Tang[1] Zhong Luo[1] Xiaowei Qiu[1] Yanke Zhang[2] Xi Lu[2] Hao huang[1] Zhongxiang Xu[1] Zucai Xu[1]

PTZ kindling model

hippocampus

GHRH

GAPDH

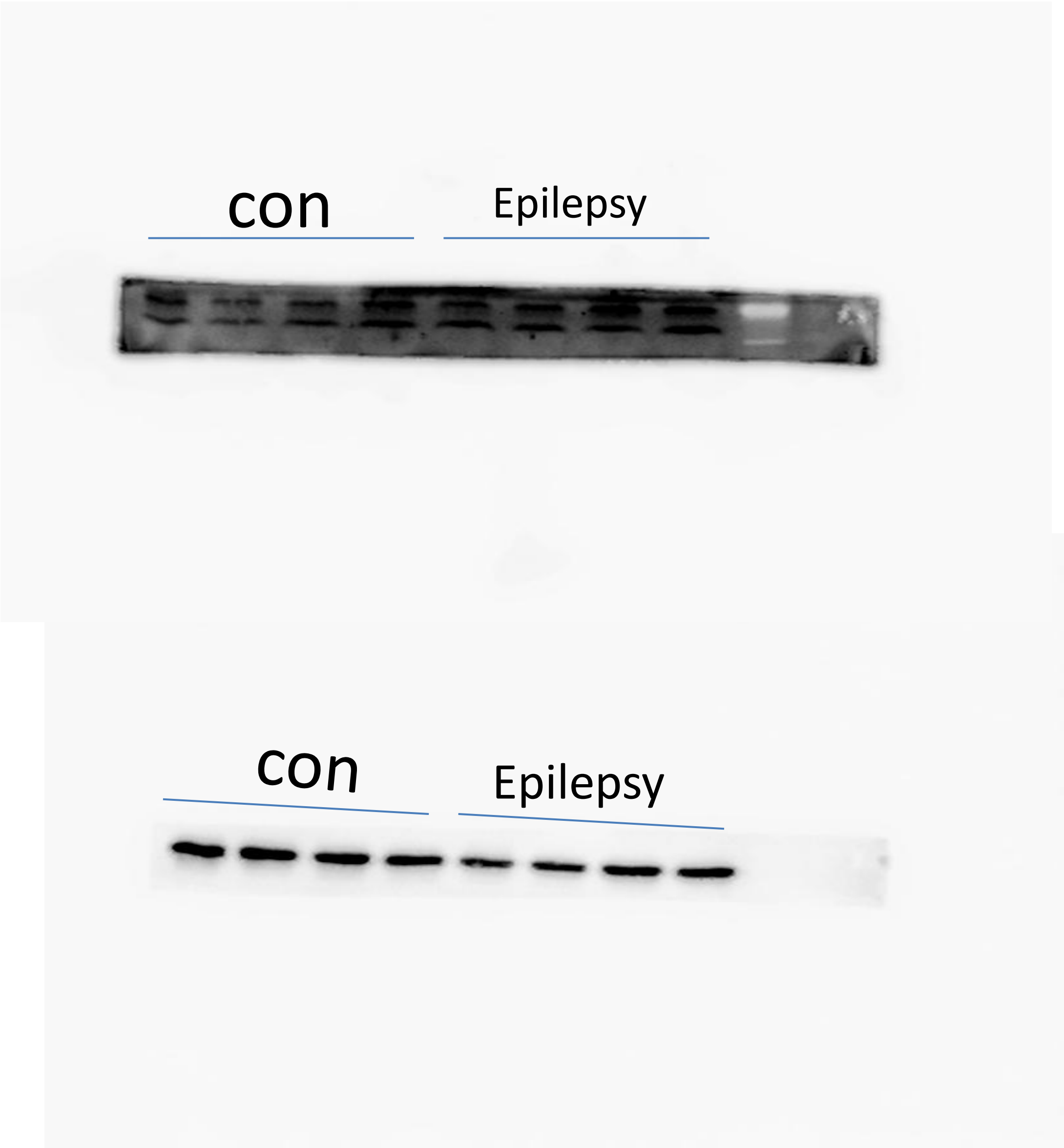

Interactions between GHRH and GABAARs in the brains of patients with epilepsy and in animal models of epilepsy

Shirong Tang[1] Zhong Luo[1] Xiaowei Qiu[1] Yanke Zhang[2] Xi Lu[2] Hao huang[1] Zhongxiang Xu[1] Zucai Xu[1]

PTZ kindling model

cortex

GHRH

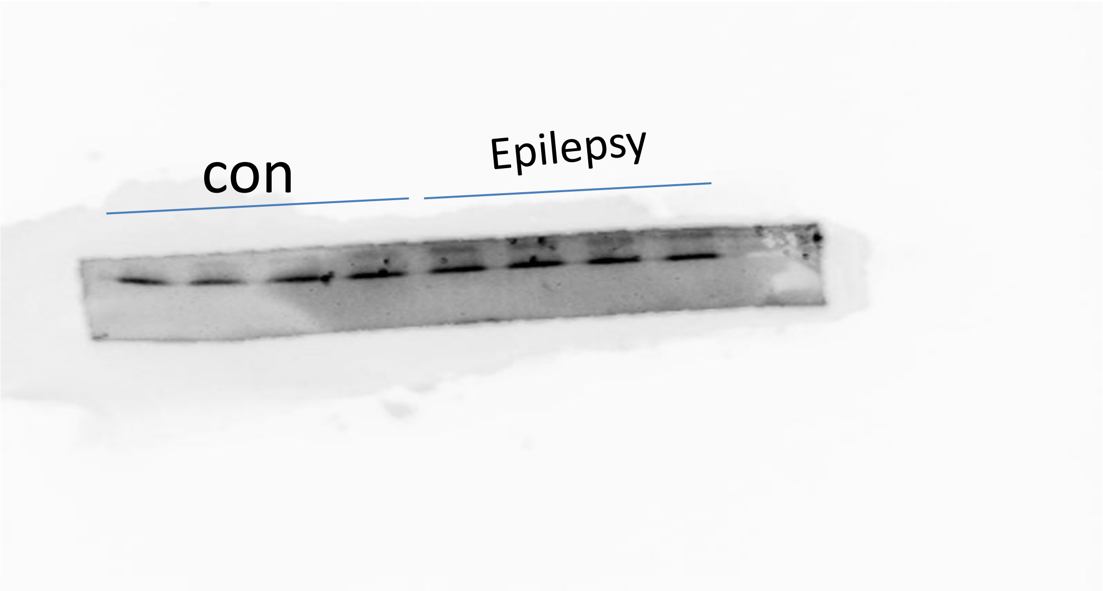

GAPDH

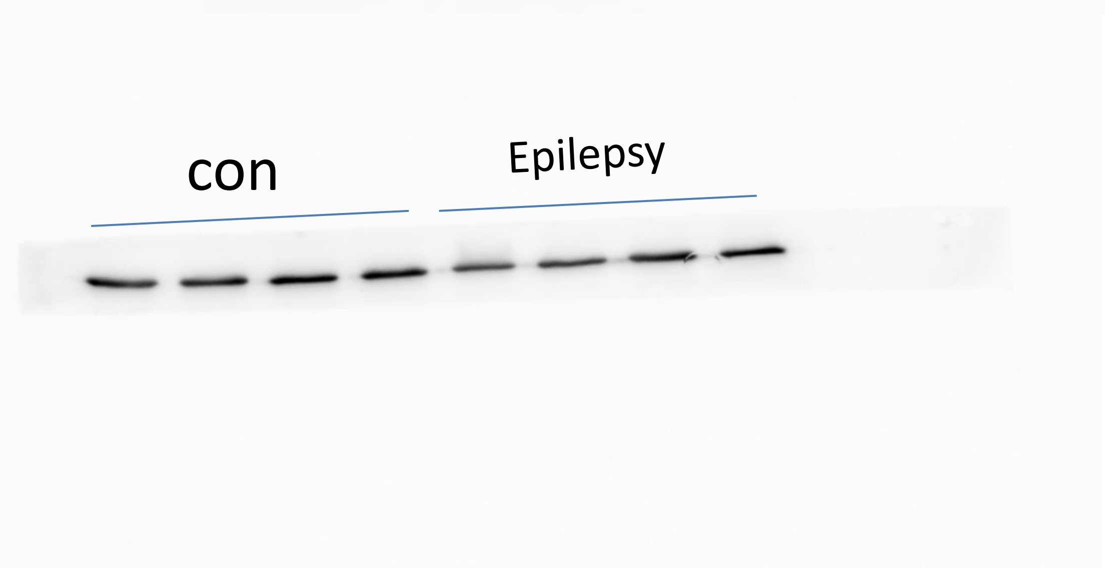

Supplement: Supplementary file 1 — Supplementary Information [file 41598_2017_18416_MOESM1_ESM.pdf]
